# Immunomodulatory dynamics in the porcine myometrium: global transcriptome analysis, including the effects of PPARγ ligands

**DOI:** 10.1186/s12864-024-11083-7

**Published:** 2024-12-05

**Authors:** Aleksandra Kurzynska, Karol Mierzejewski, Monika Golubska, Jan Pawel Jastrzebski, Iwona Bogacka

**Affiliations:** 1 Department of Animal Anatomy and Physiology, University of Warmia and Mazury in Olsztyn, Olsztyn, Poland; 2 Department of Plant Physiology, Genetics and Biotechnology, University of Warmia and Mazury in Olsztyn, Olsztyn, Poland

**Keywords:** Immune response, Cytokine, Transcription factors, Pioglitazone, T0070907

## Abstract

**Background:**

The myometrium is involved in many processes during pregnancy and the estrous/menstrual cycle. Peroxisome proliferator-activated receptors (PPARs) can be regulators of the processes occurring in the myometrium. In the present study, we determined the global transcriptome profile of the porcine myometrium during the peri-implantation period and the late luteal phase of the estrous cycle. In addition, we investigated for the first time the influence of PPARγ ligands on the transcriptome profile.

**Results:**

The myometrium of gilts (*n* = 3) was collected on days 10–11 and 14–15 of pregnancy and on the corresponding days of the estrous cycle. The expression of PPARγ was confirmed in the tissue. Based on the mRNA level, further studies were conducted on myometrial explants obtained from pigs at days 14–15 of pregnancy and the corresponding days of the estrous cycle. The tissue sections were incubated in vitro for 6 h in the presence of a PPARγ agonist, pioglitazone (P; 10 µM), or antagonist, T0070907 (T; 1 µM). To identify the transcription profile of the myometrium, RNA-Seq was performed on the NovaSeq 6000 Illumina platform. This study identified 1082 differentially expressed genes (DEGs; 609 upregulated and 473 downregulated) in the porcine myometrium on days 14–15 of pregnancy compared with the corresponding days of the estrous cycle. During pregnancy, we detected 6 and 80 DEGs related to PPARγ agonist and antagonist, respectively. During the estrous cycle, we identified 4 and 17 DEGs for P and T vs. the control, respectively.

**Conclusions:**

The results indicate that the DEGs are involved in a number of processes, including the immune response, prostaglandin synthesis, cell differentiation and communication. In addition, the role of PPARγ activity in regulating the expression of genes related to the immune response and hormone synthesis in the porcine myometrium has been demonstrated.

**Supplementary Information:**

The online version contains supplementary material available at 10.1186/s12864-024-11083-7.

## Background

The myometrium is the muscular wall of the uterus, which fulfills two main functions: it grows and stretches to provide supportive environment for the developing fetus, and it provides mechanical strength during labor [1]. Additionally, during the postpartum process, the myometrium produces an exceptionally long contraction to prevent postpartum hemorrhage by occluding flow into the sheared vessels [2]. Most studies have focused on the regulation of myometrial function during pregnancy and labor, but it should be emphasized that the myometrium is also active in nonpregnant individuals. It undergoes different types of contractions during the different phases of the menstrual cycle. First, rhythmic, ‘wave-like’ contractions, known as uterine peristalsis, occur. Second, they are responsible for emptying or discharging the uterine contents, i.e., menstrual blood, assisting in sperm transport or retaining iron in cases of blood loss [3].

The activity of the myometrium is subject to hormonal and immunological regulation. Progesterone and estrogen, in particular, influence the growth of the uterus, silent activity during pregnancy and preparation for labor. Other factors such as oxytocin and prostaglandins modulate the contractility and relaxation of the myometrium [4]. In addition, the myometrium acts as an immunoregulatory tissue, releasing cytokines and chemokines to modulate inflammation and leukocyte infiltration, which contributes to the onset of labor [5]. Macrophages, particularly those with M2-like anti-inflammatory phenotypes, are abundant in uterine decidual tissue and can infiltrate the myometrium to regulate uterine contractions [6]. During labor, proinflammatory cytokines induce the infiltration of leukocytes, possibly enhancing the inflammatory response [7]. In addition, there is an influx of peripheral monocytes into the myometrium and decidua after labor, which contributes to tissue homeostasis and involution [8]. Notably, most studies have focused on the late gestational period and labor, whereas the regulation of myometrial activity during the estrous/menstrual cycle and early pregnancy is poorly understood.

Despite considerable research efforts, the exact molecular pathways and regulatory factors that control myometrial function are not well understood. One of the regulators of myometrial physiology may be peroxisome proliferator-activated receptors (PPARs). To date, three PPARs isoforms, namely, PPARα, PPARβ/δ and PPARγ, have been identified. These receptors are activated by endogenous ligands such as fatty acids and derivatives of arachidonic acid as well as by synthetic ligands such as fibrates, nonsteroidal anti-inflammatory drugs or thiazolidinediones (TZDs) [9]. The importance of PPARγ in the myometrium lies in its ability to regulate metabolism and the synthesis and release of inflammatory mediators [10]. The expression of IL-1β has been detected in porcine myometrium and has been suggested to influence embryo implantation [11]. Natural agonists of PPARγ, such as 15-deoxy-Delta (12, 14)-prostaglandin J(2) (PGJ_2_), have been shown to inhibit mRNA expression of nuclear factor NF-κB (a classical transcription factor associated with inflammation) and cyclooxygenase COX-2 (an enzyme responsible for prostaglandin synthesis) [12]. Previously we confirmed by real-time PCR the effects of PPARγ ligands on the synthesis of nuclear factor κB (NF-κB) and selected cytokines in the porcine myometrium during the late luteal phase of the estrous cycle and early pregnancy. Pioglitazone and PGJ_2_, PPARγ agonists, increased NF-κB protein abundance in the tissue. These changes occurred mainly during the peri-implantation window. Interestingly, the PPARγ agonist pioglitazone decreased the mRNA and protein expression of IL-10 in porcine myometrium on days 14–15 of pregnancy, while the PPARγ antagonist T0070907 increased the mRNA expression of TNFα in this tissue both in the late luteal phase of the estrous cycle and in early pregnancy [13]. The decreased mRNA expression of the anti-inflammatory IL-10 and the increased mRNA expression of the pro-inflammatory TNFα [14] in the presence of PPARγ ligands suggest a modulatory role of PPARγ in this tissue. Another significant aspect is that the porcine myometrium is a source of androgens, 17-β estradiol, and estrone during early pregnancy and the luteal phase of the estrous cycle [15, 16]. There are currently no data describing the role of PPARγ in hormone synthesis in the myometrium, although one study has confirmed its role in pathological conditions. PPARγ activation has been shown to affect cell proliferation and inhibit the expression of genes involved in estrogen signaling pathway in human leiomyoma cells [17].

Taking into account the above premises and considering the gap in knowledge regarding the role of PPARγ ligands in the regulation of processes in the myometrium, the aim of the present study was to investigate the global transcriptome profile of cultured porcine myometrial tissue on days 14–15 of pregnancy and the corresponding days of the estrous cycle and the effects of PPARγ ligands on transcriptome changes in the tissue during the tested phases.

## Methods

### Animals

The experimental material was collected on the basis of the recommendations of the Animal Ethics Committee of the University of Warmia and Mazury in Olsztyn, Poland. The study was conducted on crossbred pigs (Large White × Polish Landrace) from a commercial farms in Miliszewice and Biskupiec. For the preliminary studies, the animals (100 kg, 7 months old) were divided into four groups: pregnant pigs [days 10–11 (*n* = 3, maternal recognition of pregnancy) and 14–15 (*n* = 3, initial phase of implantation)] and pigs in the estrous cycle [days 10–11 (*n* = 3, mid luteal phase) and 14–15 (*n* = 3, late luteal phase)]. The main in vitro experiment was conducted on days 14–15 of pregnancy and days 14–15 of the estrous cycle. The estrous cycle of pigs was observed daily for estrous behavior in the presence of an intact boar. The day of onset of the second estrous cycle was defined as day 0 of the estrous cycle. The phase of the estrous cycle was also confirmed in the laboratory on the basis of the morphology of the ovaries [18]. Pregnant pigs were inseminated twice, on days 1 to 2 of the estrous cycle. The day after the first insemination was considered as the first day of pregnancy. In addition, pregnancy was confirmed by the presence of conceptions in both uterine horns [19]. The material was obtained from gilts intended for commercial slaughter and meat processing. Pigs were stunned by electricity (electronarcosis) and bled to death by cutting the carotid artery (exsanguination) in the commercial slaughter, according to European legislation (EFSA, AHAW/04–027). According to the Polish Act of 15 January 2015 (Journal of Laws, 2015, item 266) and the European Parliament Act of 22 September 2010 (Directive 2010/63/EU) on the protection of animals used for scientific or educational purposes, the experiments did not require the consent of the competent ethics committee for animal experiments. The study was designed in accordance with ARRIVE guidelines. After slaughter, the dissected uteri were transported to the laboratory on ice in phosphate-buffered saline (PBS) supplemented with the following antibiotics: 100 IU/mL penicillin and 100 mg/mL streptomycin (PolfaTarchomin, Poland).

### In vitro experiment

The procedure for the collection and incubation of porcine myometrial tissue has already been described [13]. In the laboratory, the middle part of the uterine horns was separated, and then opened longitudinally with scissors at the mesometrial surface and the entire layer of myometrium was separated from the endometrium and perimetrium by careful pulling with forceps and scraping with a scalpel blade. Small fragments of the myometrium were then cleaned and placed on ice in a sterile Petri dish. The tissue sections (200 ± 10 mg, 3 mm thick) were incubated in M199 medium (Sigma Aldrich, St. Louis, MO, USA) supplemented with 0.1% BSA fraction V (Sigma Aldrich), the antibiotic nystatin (120 IU/mL; Sigma Aldrich) and gentamicin (40 mg/mL; Sigma Aldrich). The explants were preincubated for 2 h in a water bath at 37 °C in an atmosphere of 95% O_2_ and 5% CO_2_. The medium was then removed and the explants were incubated with PPARγ ligands for 6 h: pioglitazone (P; synthetic agonist; 10 µM; Cayman Chemical Company) or T0070907 (T; antagonist; 1 µM; Cayman Chemical Company). The duration of incubation with the treatments and their dosages were determined in our preliminary experiments and other studies on the endometrium and corpus luteum of pigs [20–23]. The controls (Ctr; untreated explants) contained culture medium and DMSO (dimethyl sulfoxide, an organic solvent for the tested PPARγ ligands). After incubation, the tissue explants were washed with PBS and frozen at − 80 °C to isolate total RNA. The sections were stored until RNA-Seq and real-time PCR analysis.

### RNA isolation, library preparation and sequencing procedure

For the preliminary studies, total RNA was isolated from 12 samples (4 groups × 3 pigs) using the “Total RNA Mini Kit” (A&A Biotechnology, Poland). For the main experiment, total RNA was isolated from 18 samples (2 groups × 3 pigs × 3 treatments) using the “RNeasy Fibrous Tissue Mini Kit” (Qiagen, Germany) according to the manufacturer’s protocol. The concentration and purity of the isolated RNA were measured via a Tecan Infinite M200 plate reader (Tecan Group Ltd., Switzerland). The degradation of the sample was assessed via an Agilent Bioanalyzer 2100 (Agilent Technology, USA). Eighteen RNA samples with an RNA integrity number (RIN) > 7 were selected for further analysis. The procedure for library preparation and sequencing procedures has been described previously [24]. Briefly, the TruSeq Stranded mRNA LT Sample Prep Kit (Illumina, San Diego, CA, USA) was used for library preparation. The genetic material was fragmented and the RNA was then transcribed into cDNA via reverse transcriptase. The fragments of double-stranded cDNA were labeled with specific adapters for each library. The constructed portions of the cDNA were strand specific. Finally, the pooled libraries were transferred for sequencing on the Illumina NovaSeq 6000 platform with 2 × 150 bp paired-end (PE) chemistry.

### Quality controls and mapping to the genome

The quality of the raw paired-end reads was controlled via FastQC, and the sequences were processed such that they were at least > 120 bp long, had a PHRED score of > 20 and were trimmed to the same length via Trimmomatic. High-quality trimmed reads were aligned to the Sus_scrofa 11.1 genome assembly with reference ENSEMBL annotation (version 98) via spliced transcript alignment to a reference (STAR) aligner. The mapping results were indexed and sorted by coordinates. Gene expression values (read counts) were reconstructed by compiling ballgown and the prepDE.py script. The sequencing data (PRJEB75250) were submitted to the European Nucleotide Archive (ENA).

### Differentially expressed genes

Analysis of differentially expressed genes (DEGs) and the corresponding false discovery rates (FDRs < 0.05) were performed via DESeq2. Changes in the pattern of gene expression in the porcine myometrium at days 14–15 of pregnancy and during the estrous cycle in pigs treated in vitro with the PPARγ ligands pioglitazone (P) and T0070907 (T) or without treatment (Ctr) were determined via high-throughput transcriptome sequencing. Transcriptomic effects were examined in six comparisons: Ctr on days 14–15 of pregnancy vs. Ctr on days 14–15 of the estrous cycle and P vs. Ctr and T vs. Ctr in each of the two groups. In addition, fragments per kilobase of transcript per million mapped reads (FPKM) were calculated as a normalized measure of expression that depends on sequencing depth and genomic feature length. The enrichment of key biological processes and metabolic pathways among the DEGs was identified via the enriched GO and enriched KEGG methods implemented in the ontology-based clusterProfiler R package [25]. For functional enrichment analysis, the parameters (organism, pig; ont, CC, MF, or BP; P-adjustment value cutoff, 0.05; P-adjustment method, BH) were used as cutoff criteria.

### Real-time PCR

The mRNA expression of PPARγ in porcine myometrial explants was analyzed. Primer sequences and Taqman probes for PPARγ and GAPDH were designed using Primer Express 3 Software (Applied Biosystems, CA, USA) and synthesized by Applied Biosystems. Real-time PCR was conducted in duplicate for each sample using the 7300 real-time PCR system (Applied Biosystems, USA) according to previously established protocols [26]. Differentially expressed genes were validated by real-time PCR via the AriaMx real-time PCR System (Agilent Technology, USA) as previously described [18]. Primer and probe sequences (Supplemental Table S4) for the reference (β-ACTIN and GAPDH) and target genes (PPARγ, IL6, IDO1, NR4A3, TNFAIP3, CCL5, and KDR) were designed via Primer-BLAST software (National Library of Medicine, USA). The PCR mixture, with a final volume of 25 µl, consisted of cDNA (4 ng), 300 µM primers, 12.5 µl of Power SYBR Green PCR Master Mix (Applied Biosystems, USA), and RNase-free water. Standard curves for the tested genes were generated via serial dilution of a known amount of total cDNA (from 100 to 0.032 ng of cDNA). Analyses were performed in duplicate for each sample. The non template control (NTC) was included in each measurement. Data related to the DEGs validation were presented as a fold change in gene expression, normalized to endogenous reference genes (β-ACTIN and GAPDH) and relative to the untreated control (relative quantification RQ = 1). These calculations were performed using the comparative Pfaffl method [20]. The obtained results of mRNA expression were analyzed by Student’s t-test with Welch’s correction and presented as means ± SEMs. The values were considered statistically significant at *p* < 0.05.

## Results

### PPARγ mRNA abundance

**Fig. 1:**
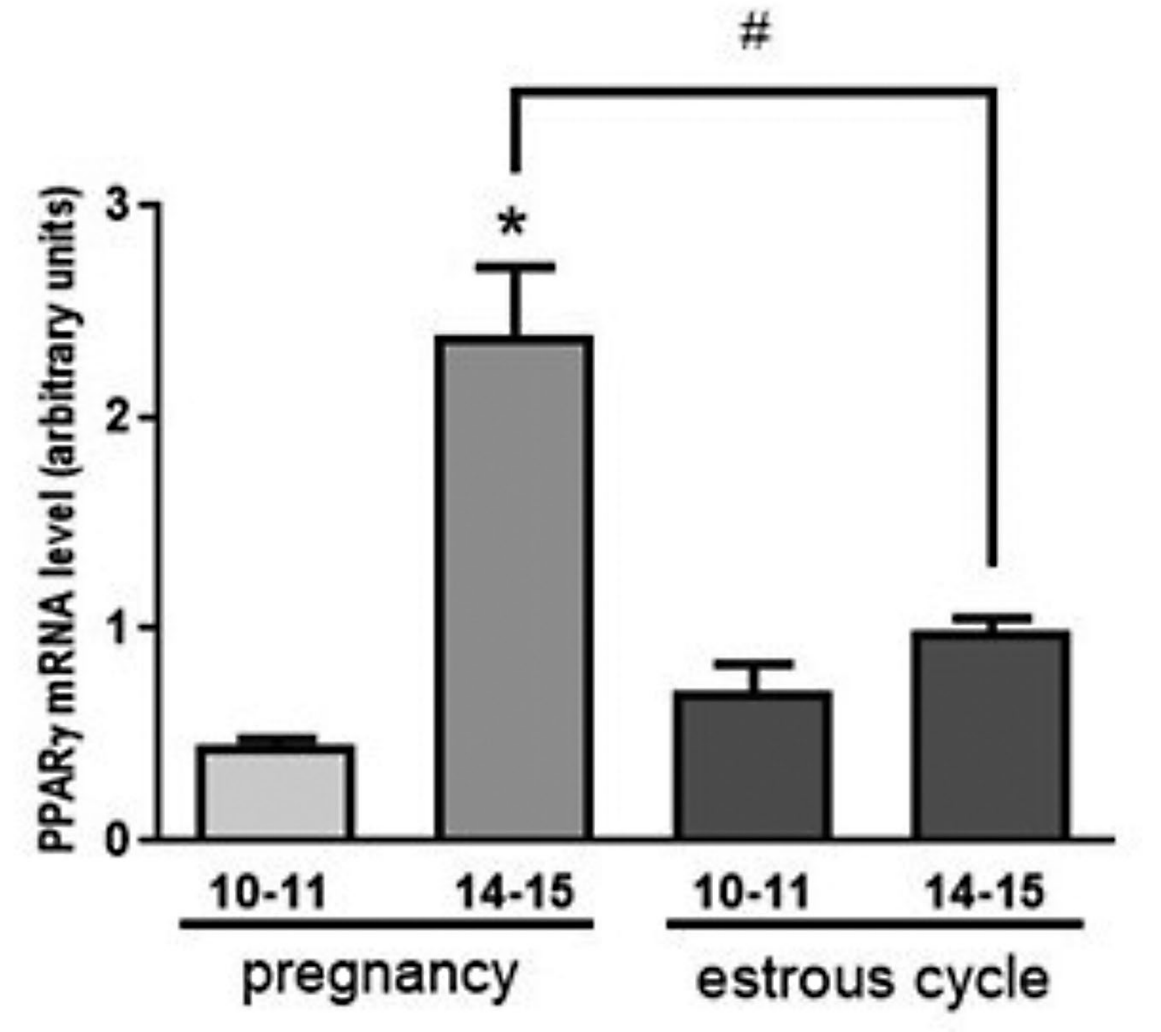
The abundance of peroxisome proliferator-activated receptor γ (PPARγ) mRNA was assessed in porcine myometrium on days 10–11 and 14–15 of pregnancy and the corresponding days of the estrous cycle. The mRNA expression levels were quantified using real-time PCR. Data were normalized by calculating the ratio of the target gene to the housekeeping gene, GAPDH, and expressed in arbitrary units. Bars marked with asterisks represent statistically significant differences (*P* < 0.05) within the same physiological state (pregnancy or estrous cycle). Bars marked with hashtags highlight significant differences between days 14–15 of pregnancy and the estrous cycle

The expression of PPARγ mRNA in porcine myometrial tissue on days 10–11 and 14–15 of pregnancy, as well as the corresponding days of the estrous cycle is depicted in Fig. 1. During pregnancy, PPARγ mRNA levels were significantly higher on days 14–15 compared to days 10–11. No significant changes in PPARγ mRNA abundance were observed during the analyzed periods of the estrous cycle. Additionally, when comparing PPARγ mRNA levels between the corresponding stages pregnancy and the estrous cycle, significantly higher PPARγ gene expression was noted on days 14–15 of pregnancy compared to the corresponding days of the estrous cycle.

### Statistics of RNA sequencing

RNA sequencing data were generated for 18 samples, including untreated samples (controls), samples treated with the PPARγ agonist pioglitazone at a concentration of 10 µM and samples treated with the PPARγ antagonist T0070907 at a concentration of 1 µM. The analysis revealed a total of 508,473,673 raw paired-end reads, with an average of 28,248,537 per sample. Short reads, low-quality sequences and ambiguous nucleotides were removed from the raw reads, leaving an average of 27,439,642 valid reads per sample, which were used for further analysis (Supplemental Table S1). The filtered reads were mapped to Ss11.1. version of the porcine genome, with an average rate of 24,957,497 unique mappings. Analysis of the distribution of mapped reads across gene structures revealed that 97.12% of the read pairs (on average per sample) were mapped to coding sequences, and 97.12% of the trimmed reads were used for further analysis. We obtained uniquely mapped reads with an average of 24,957,497, which corresponded to 90.83% (on average per sample) of the coding sequences. The RNA-seq data were deposited in the European Nucleotide Archive (ENA) database under accession number PRJEB75250.

### Comparison of the myometrial transcriptomes of pigs on days 14–15 of pregnancy and the corresponding days of the estrous cycle

**Fig. 2:**
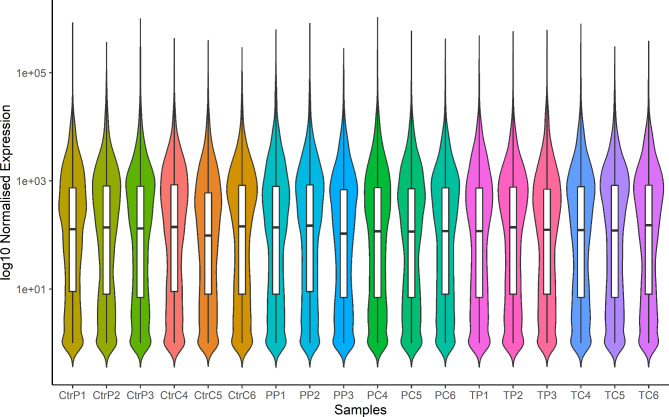
Violin plot shows the distribution of gene expression levels in the porcine myometrium. Samples incubated for 6 h with culture medium (control, **Ctr**) or culture medium with pioglitazone (10 µM, **P**) or T0070907 (1 µM, **T**) in two experimental groups: pigs on days 14–15 of pregnancy (denoted by the last letter **P**) or on days 14–15 of the estrous cycle (denoted by the last letter **C**). Numbers 1–3 refer to the biological replicates in the group on days 14–15 of pregnancy, whereas numbers 4–6 refer to the biological replicates in the group on days 14–15 of the estrous cycle. Wide sections of the violins indicate a high density of data points with the white rectangles indicating the median of the expression values and the black bars indicating the interquartile range

**Fig. 3:**
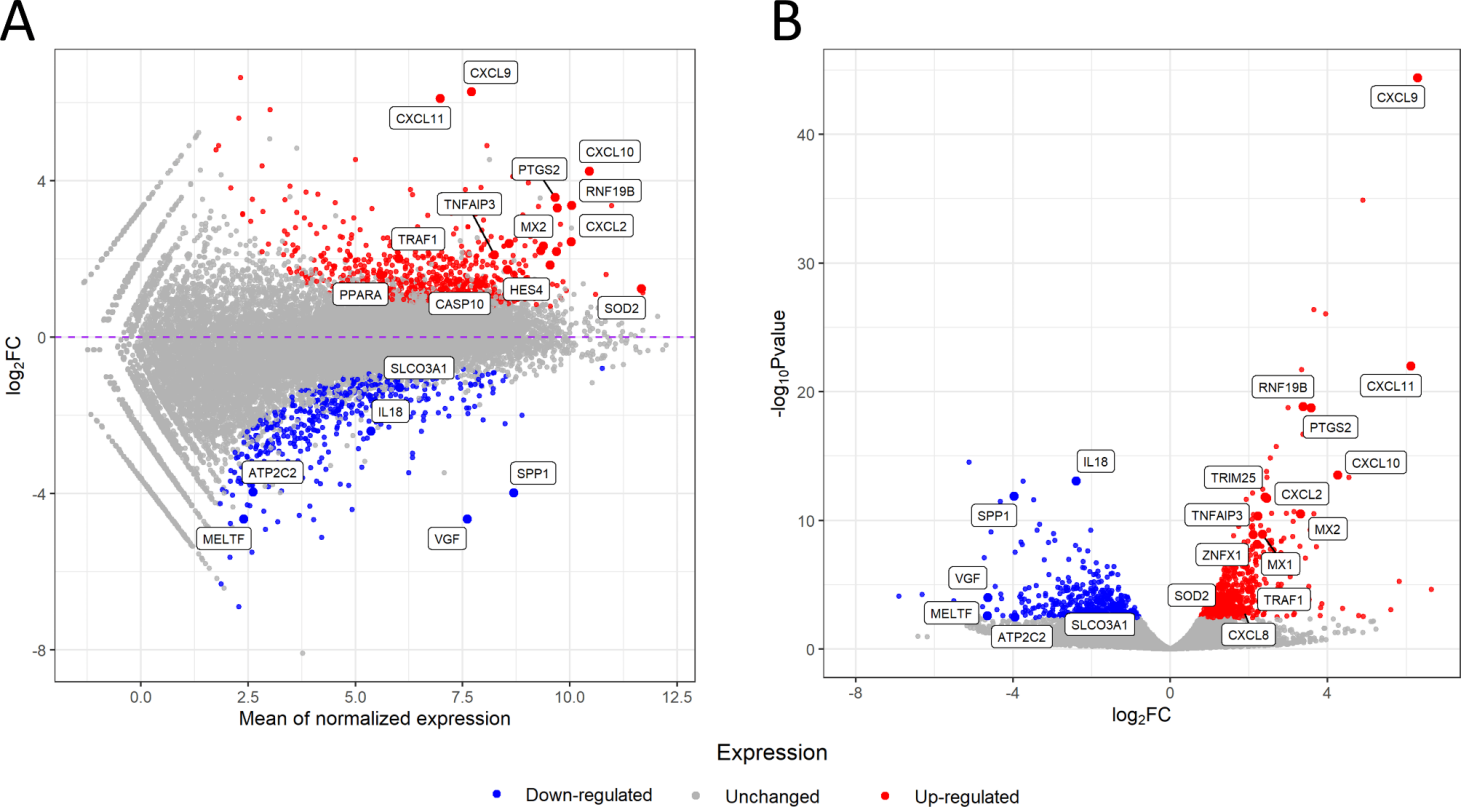
MAplot and Volcano plot shows the differences in gene expression in the porcine myometrium. **(A)** MAplot shows the logarithmic scale of the fold changes on the Y-axis and the normalized expression values on the X-axis. **(B)** Volcano plot shows the relationship between the logarithmic adjusted P-values on the Y-axis and the logarithmic scale of the fold changes on the X-axis. Differentially expressed genes are marked in blue (downregulated) and red (upregulated). The grey dots indicate genes whose expression did not change compared with that of the control

**Fig. 4:**
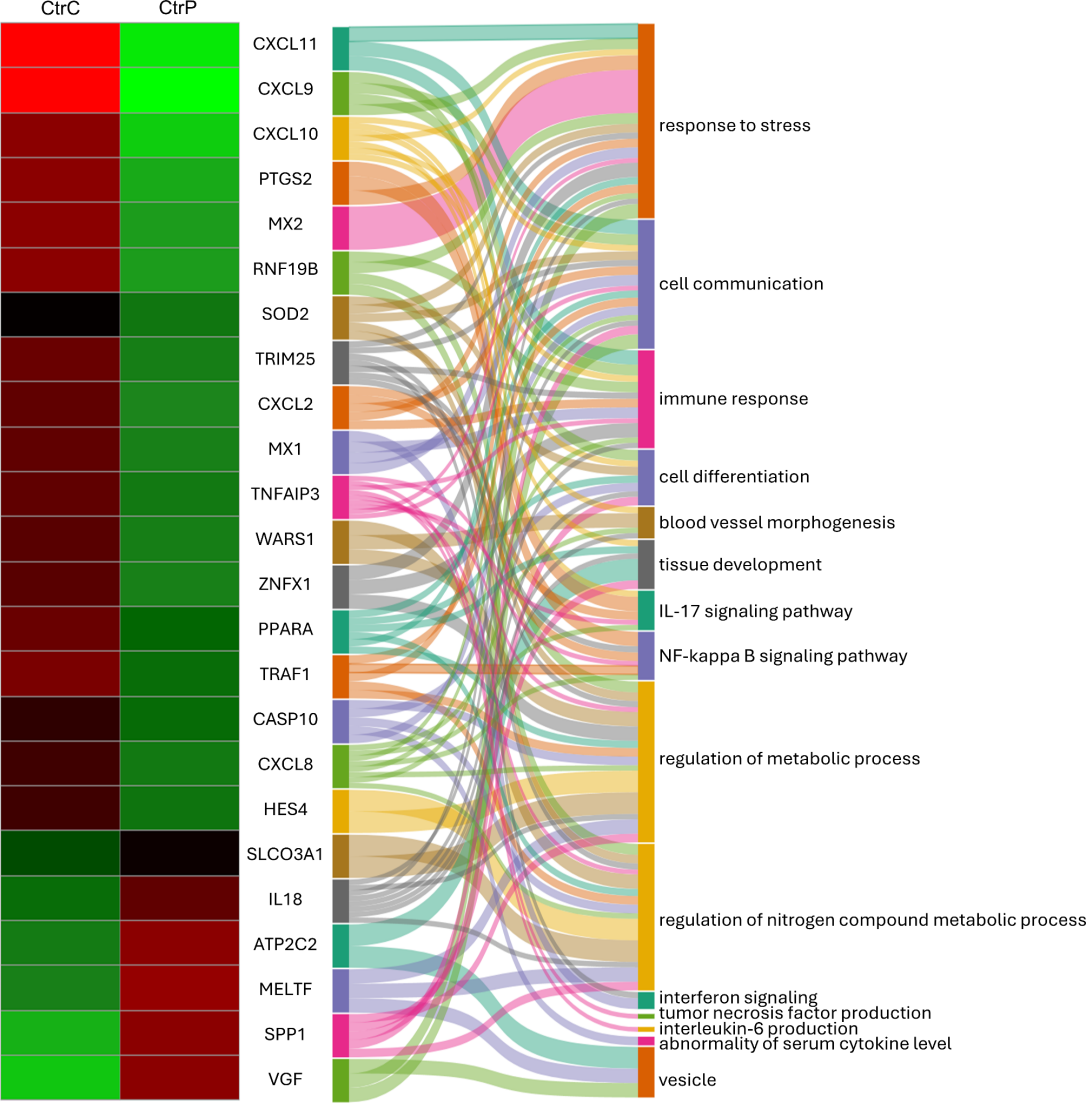
Heatmap presenting the expression of differentially expressed genes (DEGs) in the porcine myometrium. DEGs in the porcine myometrium on days 14–15 of pregnancy (**CtrP**) vs. days 14–15 of the estrous cycle (**CtrC**) and the interplay between DEGs and related biological processes or pathways in the Sankey diagram. Both columns on the heatmap show the Z-scores of gene expression (red: upregulated genes; green: downregulated genes), while the link widths represent the strength of the gene–process relationships

The analyzed samples showed a symmetrical distribution with consistency in the central portion of the data across all groups (Fig. 2). RNA-Seq analysis revealed 1082 DEGs (609 upregulated and 473 downregulated) in the porcine myometrium on days 14–15 of pregnancy compared with days 14–15 of the estrous cycle (Fig. 3). Gene Ontology (GO) analysis assigned these DEGs to 356 terms related to biological processes, 21 terms related to cellular components, and 46 terms related to molecular functions. Furthermore, these DEGs were assigned to 13 KEGG, 13 HP and 4 REAC pathways (Supplemental Table S2). The differentially expressed genes were involved in processes such as the regulation of metabolic processes (358 DEGs, including *EDN1*, *SMAD7* and *SLCO3A1*), cell communication (356 DEGs, including *CXCL10*,* RHEBL1*,* and C1QL4*), the regulation of nitrogen compound metabolic processes (306 DEGs, including *SPP1*, *SOD2*, and *HES4*), response to stress (257 DEGs, including *MX2*, *CXCL11*, and *CXCL9*), cell differentiation (200 DEGs, including *PPARA*,* C1QL4*,* and FAIM2*), immune response (146 DEGs, including *NFKBIA*, *RNF19B*, and *ZNFX1*), vesicle (111 DEGs, including *VEGF*, *MELTF*, and *ATP2C2*), tissue development (100 DEGs, including *IL18*, *ATP2C2*,* and DDR1*), blood vessel morphogenesis (40 DEGs, including *CXCL8*,* RUNX2*,* and STRA6*), interleukin-6 production (17 DEGs, including *TNFAIP3*,* SLAMF1*,* and P2RX7*) and tumor necrosis factor production (17 DEGs, including *THBS1*,* JAK2*,* and ANGPT1*). Moreover, KEGG enrichment analysis revealed that DEGs were involved in signaling pathways such as the TNF signaling pathway (16 DEGs, including *FAS*,* ICAM1*,* and IRF1*), the NF-kappa B signaling pathway (17 DEGs, including *PTGS2*,* TRAF1*,* and TNFSF13B*) and the IL17 signaling pathway (14 DEGs, including *CXCL2*,* MAPK6*,* and Table 2*). In turn, HP enrichment analysis revealed that DEGs were involved in signaling pathways such as abnormalities in temperature regulation (51 DEGs, including *PSMB9*,* DAAM2*,* and PML*), abnormal leukocyte count (48 DEGs, including *FUT8*,* MAD2L2*,* and STX11*) or abnormalities in serum cytokine levels (11 DEGs, including *STX11*,* ADAR*, and *CASP10*). In addition, REAC enrichment analysis revealed that the DEGs were involved in signaling pathways such as interferon signaling (16 DEGs, including *EIF4G3*, *MX1*, and *TRIM25*) or antiviral mechanisms involving IFN-stimulated genes (11 DEGs, including *OASL*,* PDE12*,* and RNASEL*). The selected DEGs and their interplay with related biological processes or pathways are presented in Fig. 4. All detailed DEGs are described in Supplemental Table S3, while the GO, KEGG, HP and REAC results are described in Supplemental Table S2.

### Effects of the PPARγ agonist pioglitazone on the global transcriptome profile of the porcine myometrium

**Fig. 5:**
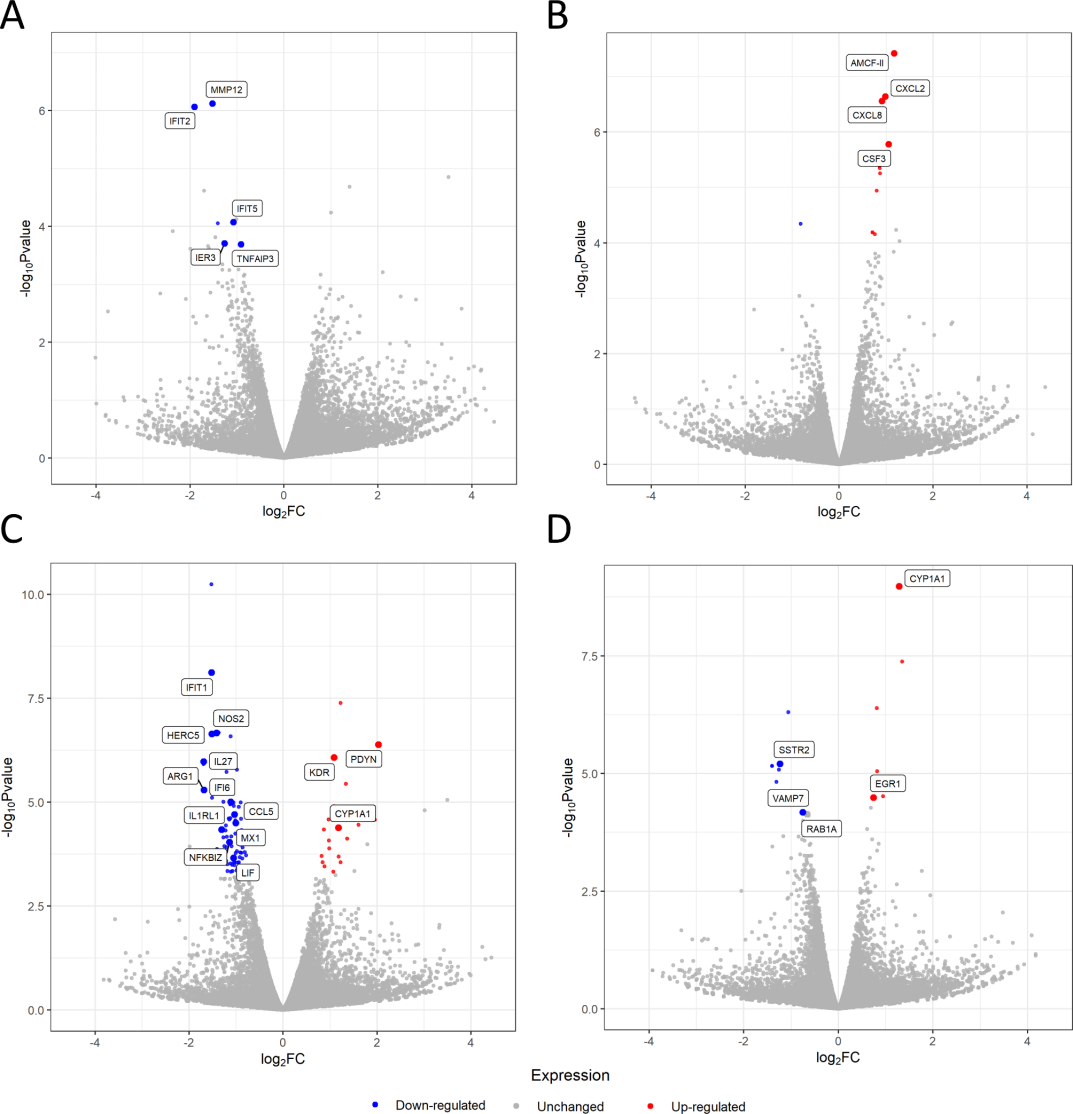
Volcano plots. The logarithmic adjusted P-values are present on the Y-axis and the logarithmic scale of fold changes on the X-axis. Differentially expressed genes are marked in blue (downregulated) and red (upregulated). The grey dots indicate genes whose expression did not change compared with that of the control. Pioglitazone (10 µM) vs. control treatment of the myometrium of gilts on days 14–15 of pregnancy (**A**) and days 14–15 of the estrous cycle (**B**). T0070907 (1 µM) vs. control treatment of the myometrium of pigs on days 14–15 of pregnancy (**C**) and days 14–15 of the estrous cycle (**D**)

The influence of pioglitazone on the gene expression profile of the porcine myometrium on days 14–15 of pregnancy and the corresponding days of the estrous cycle was determined. Pioglitazone downregulated the expression of six genes (*TNFAIP3*,* NR4A3*,* IFIT2*,* IFIT5*,* MMP12*,* and IER3*) on days 14–15 of pregnancy (Fig. 5A; Table 1) and upregulated the expression of four genes (*CXCL8*,* AMCF-II*,* CXCL2*,* and CSF3*) on days 14–15 of the estrous cycle (Fig. 5B; Table 1).

**Table 1:** DEGs identified in the porcine myometrium after treatment with the PPARγ agonist pioglitazone

Gene name	Row name	log2FoldChange		BaseMean
**Days 14–15 of pregnancy**				
*TNFAIP3*	ENSSSCG00000004154		-0.92	5413.11
*NR4A3*	ENSSSCG00000005385		-1.41	8787.17
*IFIT2*	ENSSSCG00000010451		-1.91	6242.64
*IFIT5*	ENSSSCG00000010454		-1.08	10461.44
*MMP12*	ENSSSCG00000014987		-1.53	5922.26
*IER3*	ENSSSCG00000027607		-1.27	8945.60
**Days 14–15 of the estrous cycle**				
*CXCL8*	ENSSSCG00000008953		0.91	9271.49
*AMCF-II*	ENSSSCG00000008957		1.17	3890.52
*CXCL2*	ENSSSCG00000008959		0.98	10945.15
*CSF3*	ENSSSCG00000017488		1.05	418.46

Footnote: DEGs: differentially expressed genes; *TNFAIP3*: TNF alpha-induced protein 3; *NR4A3*: nuclear receptor subfamily 4 group A member 3; *IFIT2*: interferon-induced protein with tetratricopeptide repeats 2; *IFIT3*: interferon-induced protein with tetratricopeptide repeats 3; *MMP12*: matrix metallopeptidase 12; *IER3*: immediate early response 3; *CXCL8*: C-X-C motif chemokine ligand 8; *AMCF-II*: alveolar macrophage-derived chemotactic factor-II; *CXCL2*: chemokine (C-X-C motif) ligand 2; *CSF3*: colony stimulating factor 3)

**Fig. 6:**
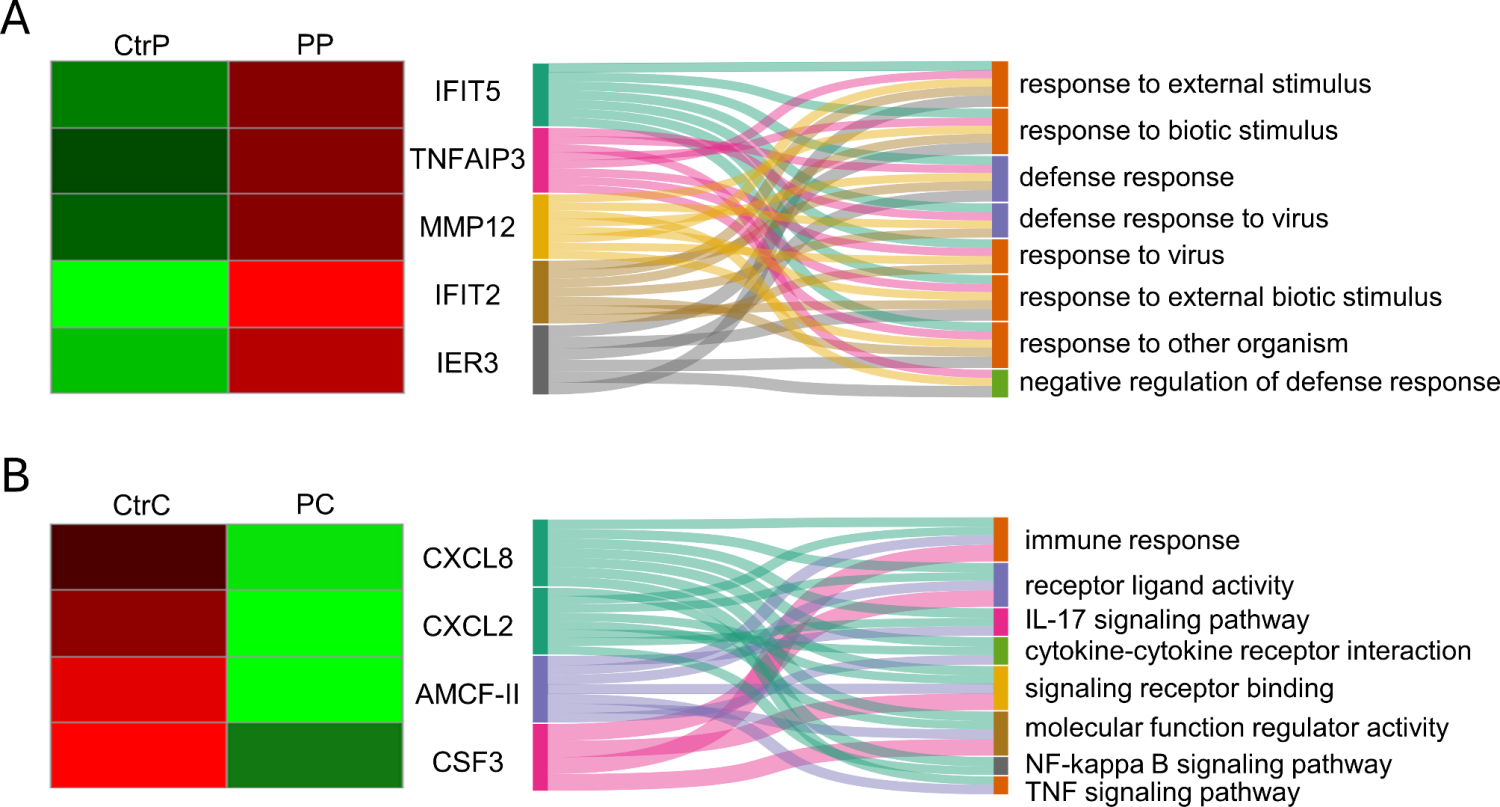
Selected DEGs and associated processes following treatment of porcine myometrium with the PPARγ agonist pioglitazone. Heatmap presenting the expression of differentially expressed genes (DEGs) in the porcine myometrium treated with pioglitazone (10 µM, P) vs. the control group (Ctr) on days 14–15 of pregnancy (denoted by the last letter P, diagram **A**) or on days 14–15 of the estrous cycle (denoted by the last letter C, diagram **B**) and the interplay between DEGs and related biological processes in the Sankey diagram. Both columns on the heatmap show the Z-scores of gene expression (red: upregulated genes; green: downregulated genes), while the link widths represent the strength of the gene–process relationships

According to the GO analysis, the DEGs in the porcine myometrium on days 14–15 of pregnancy were associated with, for example, the defense response to viruses, the response to other organisms, the response to external biotic stimuli, the response to biotic stimuli, the defense response, the negative regulation of the defense response or the response to external stimuli (Fig. 6A). The altered genes, *CXCL8*, *AMCF-II*, *CXCL2* and *CSF3*, are involved in processes such as the immune response, receptor ligand activity, cytokine‒cytokine receptor interaction, signaling receptor binding and molecular function regulator activity. Moreover, KEGG enrichment analysis revealed that the DEGs were involved in signaling pathways such as the IL-17 signaling pathway, NF-kappa B signaling pathway and TNF signaling pathway (Fig. 6B). In addition, REAC enrichment analysis revealed that the DEGs were involved in the pathway of chemokine receptor binding to chemokines. All detailed DEGs and the GO, KEGG, and REAC results are described in Supplemental Tables S2 and S3, respectively.

### Effects of the PPARγ antagonist T0070907 on the global transcriptome profile of the porcine myometrium

The influence of T0070907 on the gene expression profile of the porcine myometrium on days 14–15 of pregnancy and the corresponding days of the estrous cycle was determined. T0070907 downregulated the expression of 64 genes (e.g., *IL27*,* ARG1*,* and NOS2*) and upregulated the expression of 16 genes (e.g., *KDR*,* CYP1A1*,* and PDYN*) on days 14–15 of pregnancy (Fig. 5C; Table 2). In turn, on days 14–15 of the estrous cycle, the antagonist downregulated the expression of 11 DEGs (e.g., *RAB1A*,* SSTR2*,* and VAMP7*) and upregulated the expression of 6 genes (e.g., *CYP1A1*,* EGR1 and MMP9*) (Fig. 5D Table 2).

**Table 2:** DEGs identified in the porcine myometrium after treatment with the PPARγ antagonist T0070907

Gene name	Row name	log2FoldChange		BaseMean
**Days 14–15 of pregnancy**				
**Three selected downregulated DEGs**				
*IL27*	ENSSSCG00000039300		-1.70	207.72
*ARG1*	ENSSSCG00000004195		-1.69	589.15
*NOS2*	ENSSSCG00000017755		-1.42	642.40
***Three selected upregulated DEGs***				
*PDYN*	ENSSSCG00000037684		2.03	155.82
*CYP1A1*	ENSSSCG00000001906		1.18	377.06
*KDR*	ENSSSCG00000008844		1.08	1579.08
**Days 14–15 of the estrous cycle** ***Three selected downregulated DEGs***				
*SSTR2*	ENSSSCG00000017249		-1.24	165.77
*VAMP7*	ENSSSCG00000029615		-0.75	587.64
*RAB1A*	ENSSSCG00000008359		-0.66	2159.52
***Three selected upregulated DEGs***				
*MMP9*	ENSSSCG00000007436		1.35	241.73
*CYP1A1*	ENSSSCG00000001906		1.05	418.46
*EGR1*	ENSSSCG00000014336		0.74	15415.89

Footnote: DEGs: differentially expressed genes; *IL27*: interleukin 27; *ARG1*: arginase 1; *NOS2*: nitric oxide synthase 2; *PDYN*: prodynorphin; CYP1A1: cytochrome P450 family 1 subfamily A member 1; *KDR*: kinase insert domain receptor; *SSTR2*: somatostatin receptor 2; *VAMP7*: vesicle associated membrane protein 7; *RAB1A*: RAB1A, member RAS onco family; *MMP9*: matrix metallopeptidase 9; *EGR1*: early growth response 1.

**Fig. 7:**
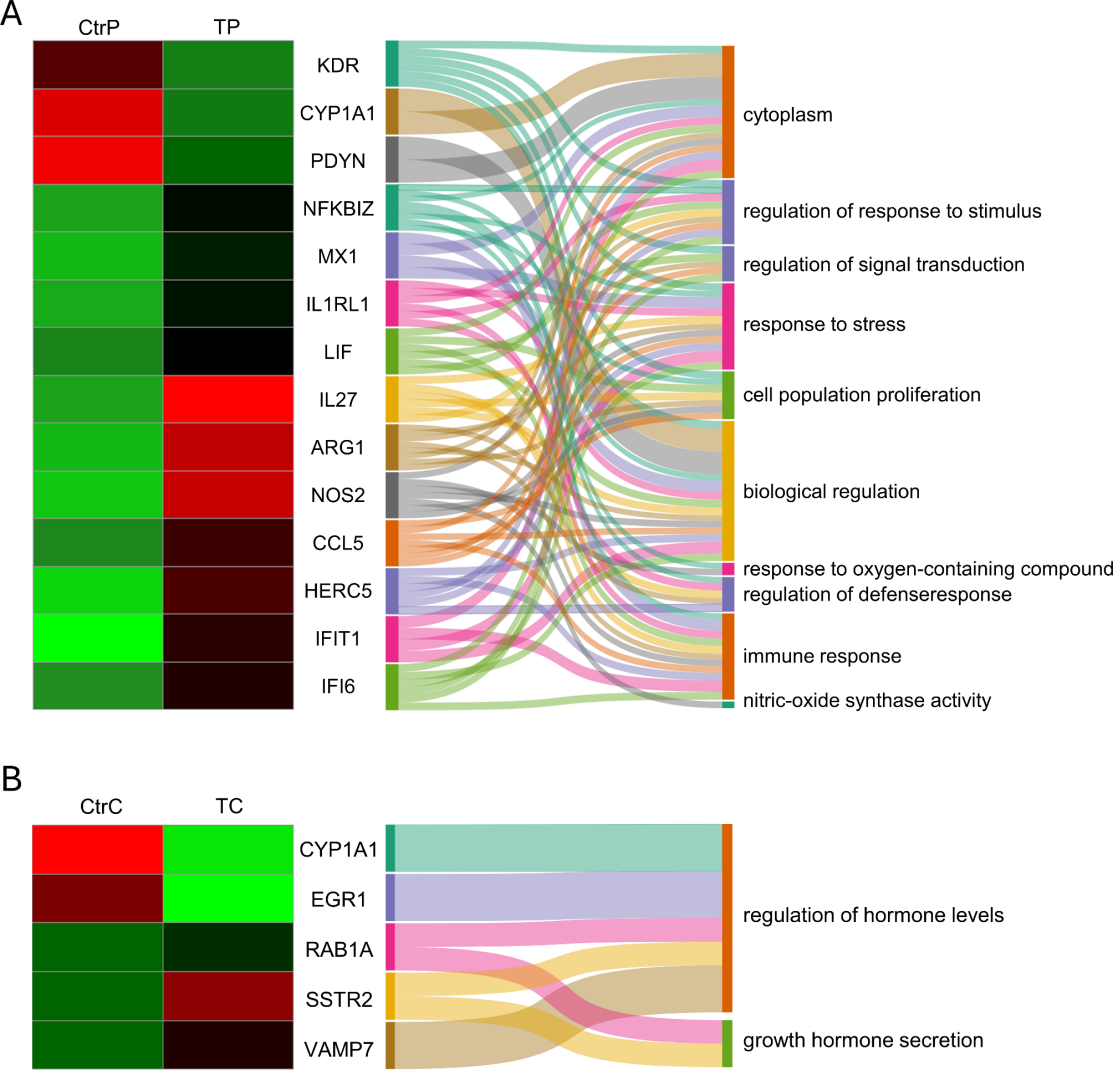
Selected DEGs and associated processes following treatment of porcine myometrium with the PPARγ antagonist T0070907. Heatmap presenting the expression of differentially expressed genes (DEGs) in the porcine myometrium treated with T0070907 (1 µM, T) vs. the control group (Ctr) on days 14–15 of pregnancy (denoted by the last letter P, diagram **A**) or on days 14–15 of the estrous cycle (denoted by the last letter C, diagram **B**) and the interplay between DEGs and related biological processes in the Sankey diagram. Both columns on the heatmap show the Z-scores of gene expression (red: upregulated genes, green: downregulated genes), while the link widths represent the strength of the gene–process relationships

According to the GO analysis, DEGs in the porcine myometrium on days 14–15 of pregnancy were associated with processes such as the regulation of the response to stimulus, the regulation of signal transduction, the response to stress, cell population proliferation, biological regulation, the response to oxygen-containing compounds, the regulation of the defense response, the immune response and nitric oxide synthase activity, and changes in the cytoplasm (Fig. 7A). The DEGs *detected* in the porcine myometrium on days 14–15 of the estrous cycle were involved in processes including the regulation of hormone levels and growth hormone secretion (Fig. 7B). All detailed DEGs and the GO, KEGG, and REAC results are described in Supplemental Tables S2 and S3, respectively.

## Discussion

The myometrium is the layer of smooth muscle in the uterus and is responsible for uterine contractions. The activity of the myometrium has been shown to change in pregnant and nonpregnant uteri [27]. Most studies have focused on the activity of the myometrium during labor, when it is responsible for the strong uterine contractions that facilitate the passage of the fetus through the cervix and vaginal canal [28]. It should be emphasized that the myometrium may have an indirect impact on processes occurring in the uterus and influence the success of embryo implantation [29, 30]. However, the molecular mechanisms that occur in the smooth muscle of the uterus during the peri-implantation phase are not well understood. In the present study, 1082 genes were identified that are differentially expressed in the myometrium of pigs on days 14–15 of pregnancy compared with the corresponding days 14–15 of the estrous cycle. Among these genes, 609 were upregulated, and 473 were downregulated. Notably, the DEGs in the porcine myometrium were responsible for cell differentiation and communication, prostaglandin synthesis, the immune response and metabolic regulation. In view of the large number of results obtained, we would like to concentrate on the most interesting genes and processes.

Most research has focused on the contractile properties of the myometrium [2, 3, 31]. In this study we observed the upregulation of prostaglandin endoperoxide synthase 2 *(PTGS2*) in the porcine myometrium during early pregnancy. PTGS2, also known as cyclooxygenase 2 (COX-2), is an enzyme involved in the synthesis of prostaglandins (PGs), which are crucial hormones involved in the regulation of reproductive processes [32–34]. Prostaglandins play essential roles in the induction of uterine contractility during menstruation [35] and support embryo implantation [36] and parturition [37]. We hypothesize that the diverse expression of genes related to uterine contractility is important for the successful implantation of the embryo and the maintenance of pregnancy. This hypothesis is supported by another observation – the increased expression of endothelin 1 *(EDN1)* in the porcine myometrium on days 14–15 of pregnancy. Endothelin 1 plays a role in steroidogenesis, folliculogenesis and ovulation [38] and has been defined as one of the major vasoactive factors expressed in the uterus [39]. Endothelin 1 has been found to increase Ca2 + levels and induce contraction of rat uterine smooth muscle [40]. In addition, increased *EDN1* expression in the endometrium in early pregnancy could contribute to the regulation of progesterone production and further sensitization to contraction factors. Moreover, we observed a downregulation of the expression of secreted phosphoprotein 1 *(SPP1)* in the porcine myometrium on days 14–15 compared with the corresponding days of the estrous cycle. SPP1 is a glycoprotein involved in cell communication and plays a crucial role in the attachment of the blastocyst to the endometrium. It has been reported that SPP1 knockout mice have a lower pregnancy rate [41]. Cao et al. [42] reported decreased expression of *SPP1* in the mouse myometrium on days 1–4 of gestation, which may result in decreased myometrial smooth muscle activity. These findings indicate that the expression of genes related to porcine myometrial activity changes early in pregnancy, whereas most studies have concentrated primarily on myometrial contractile activity during labor.

It should be emphasized that the immune response of the myometrium changes during pregnancy compared with the estrous cycle. We observed a downregulation of interleukin 18 *(IL18)* expression on days 14–15 of pregnancy. IL18 is a cytokine involved in immune processes in the uterus. It regulates the expression of various genes involved in the remodeling of the endometrium to prepare it for implantation and maintain immune tolerance during this process [43]. The role of IL18 in the myometrium is not well understood. It has been suggested that increased production of IL18 may disrupt immunological processes in the myometrium and may be involved in the development of adenomyosis [44]. Interestingly, the abundance of IL18 mRNA in the endometria of pregnant pigs was low until day 10 of gestation and then increased on days 15–17 [45]. Considering the role of IL18 in modulating the immune response during implantation, we can hypothesize that differential expression of this cytokine in the endometrium and the myometrium may be necessary to establish an immunological balance at this crucial moment of pregnancy.

In the present study, we evaluated the effects of PPARγ ligands on the global transcriptome profile of the porcine myometrium on days 14–15 of pregnancy and the corresponding days of the estrous cycle. We observed that the PPARγ agonist pioglitazone strongly affected the immune response in the porcine myometrium. For example, pioglitazone downregulated *TNFAIP3* expression in the porcine myometrium during the preimplantation period. TNFAIP3 encodes the A20 protein (also known as TNF-induced protein 3), a cytoplasmic protein that is required for the inhibition of NF-κB action by proinflammatory molecules such as IL-1β, TNFα or pathogens via the TLR pathway [46]. It has been noted that *TNFAIP3* is expressed in the human myometrium [47]. Moreover, the expression of *TNFAIP3* in the human chorioamniotic membrane was greater in patients with spontaneous labor at the time of delivery than in patients without labor [48]. The role of TNFAIP3 during early pregnancy is unknown. We speculate that the downregulation of *TNFAIP3* expression by pioglitazone may be related to NF-κB activation. Our previous results revealed a greater protein content in the myometrium after treatment with pioglitazone on days 14–15 of pregnancy [13]. Therefore, the present data are consistent with previous data and indicate the influence of PPARγ on immunomodulation in the porcine myometrium during early pregnancy. Pioglitazone also regulated the immune response on days 14–15 of the estrous cycle. For example, the expression of *CXCL8* and *CSF3* was upregulated. CXCL8 is a chemoattractant for neutrophils [49], whereas CSF3 stimulates the proliferation of immune cells [50]. In our previous studies, pioglitazone also upregulated the expression of these genes in the endometria of pigs with LPS-induced inflammation on days 18–20 of the estrous cycle [51]. Therefore, we hypothesize that pioglitazone has a stimulatory effect on immune activation in reproductive tissues. It is worth noting that increased expression of *CXCL8* and *CSF3* was observed in the myometrium during labor [8]. This change in the profile of immune mediators appears to be required for the induction of physiological inflammation and the restoration of homeostasis. This mechanism in the myometrium during the estrous cycle has not yet been described, so further studies are needed to clarify its significance.

The present study revealed the effect of the PPARγ antagonist T0070907 on the global transcriptome of the porcine myometrium on days 14–15 of pregnancy and days 14–15 of the estrous cycle. Interestingly, blockade of PPARγ by an antagonist altered the expression of several genes that regulate various processes, including biological regulation, the immune response, and processes related to hormone production. For example, it stimulated the expression of cytochrome P450 family 1 subfamily A member 1 *(CYP1A1)* in the porcine myometrium on days 14–15 of pregnancy and the corresponding days of the estrous cycle. CYP1A1 is one of the most important regulators of steroid hormone synthesis in reproductive tissues and is involved in estrogen catabolism [52]. 17β-Estradiol and estrone are metabolized by CYP1A1 mainly to 2-hydroxyestrone, but also to 4-hydroxyestrone and other metabolites [53]. We postulate that the inhibition of PPARγ activity may interfere with estrogen catabolism in the porcine myometrium by upregulating *CYP1A1* expression. In addition, T0070907 increased the expression of early growth response gene-1 *(EGR1)* in the porcine myometrium during the late luteal phase. As a transcription factor, EGR1 plays a role in the physiology and pathology of numerous organs, including the uterus. For example, EGR1, which is induced by 17β-estradiol, regulates the expression of a number of genes involved in endometrial epithelial cell remodeling during embryo implantation [54]. It should be emphasized that the importance of the porcine myometrium for steroid hormone synthesis has been confirmed [15, 16]. These results show that the inhibition of PPARγ activity may impair steroidogenesis in the myometrium. Therefore, further studies on the effects of PPARγ antagonists on this tissue should be conducted.

## Conclusions

To summarize, our study revealed that the global transcriptome of cultured myometrial tissue of the pig undergoes changes during early pregnancy compared with the late luteal phase of the estrous cycle. The differentially expressed genes are potentially involved in cell differentiation and communication as well as immune and metabolic processes. Additionally, we have demonstrated for the first time that PPARγ may play a significant role as a transcription factor in the myometrium, potentially regulating immune processes. However, since these findings were based on cultured tissues, further research is needed to confirm whether PPARγ exerts similar regulatory effects in vivo, and whether inhibition of PPARγ activity could disrupt myometrial physiology during implantation.

## Electronic supplementary material

Below is the link to the electronic supplementary material.

Supplementary Material 1

Supplementary Material 2

Supplementary Material 3

Supplementary Material 4

## Data Availability

The sequencing data (PRJEB75250) were submitted to the European Nucleotide Archive (ENA).

## Abbreviations

ADAR: Adenosine deaminase RNA specific
AMCF-II: Alveolar macrophage-derived chemotactic factor-II
ANGPT1: Angiopoietin 1
ARG 1: Arginase 1
ATP2C2: ATPase secretory pathway Ca2 + transporting 2
BP: Biological processes
C1QL4: Complement C1q like 4
CASP10: Caspase 10
CC: Cellular components
CCL5: C-C motif chemokine ligand 5
COX-2: Cyclooxygenase 2
CSF3: Colony stimulating factor 3
CXCL10: C-X-C motif chemokine ligand 10
CXCL11: C-X-C motif chemokine ligand 11
CXCL2: C-X-C motif chemokine ligand 2
CXCL8: C-X-C motif chemokine ligand 8
CXCL9: C-X-C motif chemokine ligand 9
CYP1A1: Cytochrome P450 family 1 subfamily A member 1.
DAAM2: Dishevelled associated activator of morphosis 2
DDR1: Discoidin domain receptor tyrosine kinase 1
DEG: Differentially expressed gene
DMSO: Dimethyl sulfoxide
EDN1: Endothelin 1
EGR1: Early growth response 1
EIF4G3: Eukaryotic translation initiation factor 4 gamma 3
FAIM2: Fas apoptotic inhibitory molecule 2
FAS: Fas cell surface death receptor
FDRs: False discovery rates
FPKM: Fragments per kilobase of transcript per million mapped reads
FUT8: Fucosyltransferase 8
GAPDH: Glyceraldehyde 3-phosphate dehydrogenase
GO: Gene ontology
HES4: Hes family bHLH transcription factor 4
HP: Human phenotype ontology
ICAM1: Intercellular adhesion molecule 1
IDO1: Indoleamine 2,3-dioxygenase 1
IER3: Immediate early response 3
IFIT2: Interferon-induced protein with tetratricopeptide repeats 2
IFIT3: Interferon-induced protein with tetratricopeptide repeats 3
IFIT5: Interferon-induced protein with tetratricopeptide repeats 5
IFN: Interferon
IL17: Interleukin 17
IL18: Interleukin 18
IL-1β: Interleukin 1β
IL27: Interleukin 27
IL6: Interleukin 6
IRF1: Interferon regulatory factor 1
JAK2: Janus kinase 2
KDR: Kinase insert domain receptor
KEGG: Kyoto encyclopedia of genes and genomes
MAD2L2: Mitotic arrest deficient 2 like 2
MAPK6: Mitogen-activated protein kinase 6
MELTF: Melanotransferrin
MF: Molecular function
MMP12: Matrix metallopeptidase 12
MMP9: Matrix metallopeptidase 9
MX1: MX dynamin like GTPase 1
MX2: MX dynamin like GTPase 2
NFKBIA: NFKB inhibitor alpha
NF-κB: Nuclear factor κB
NOS2: Nitric oxide synthase 2
NR4A3: Nuclear receptor subfamily 4 group A member 3
NTC: Nontemplate control
OASL: 2’-5’-oligoadenylate synthetase like
P2RX7: Purinergic receptor P2 × 7
PBS: Phosphate-buffered saline
PDE12: Phosphodiesterase 12
PDYN: Prodynorphin
PGs: Prostaglandins
PML: PML nuclear body scaffold
PPAR: Peroxisome proliferator-activated receptor
PPARA: Peroxisome proliferator activated receptor alpha
PSMB9: Proteasome 20 S subunit beta 9
PTGS2: Prostaglandin-endoperoxide synthase 2
RAB1A: RAB1A, member RAS onco family
REAC: Reactome pathway database
RHEBL1: RHEB like 1
RIN: RNA integrity number
RNASEL: Ribonuclease L
RNF19B: Ring finger protein 19B
RUNX2: RUNX family transcription factor 2
SLAMF1: Signaling lymphocytic activation molecule family member 1
SLCO3A1: Solute carrier organic anion transporter family member 3A1
SMAD7: SMAD family member 7
SOD2: Superoxide dismutase 2
SPP1: Secreted phosphoprotein 1
SSTR2: Somatostatin receptor 2
STRA6: Signaling receptor and transporter of retinol STRA6
STX11: Syntaxin 11
TAB2: TGF-beta activated kinase 1 (MAP3K7) binding protein 2
THBS1: Thrombospondin 1
TLR: Toll-like receptors
TNFAIP3: TNF alpha-induced protein 3
TNFSF13B: TNF superfamily member 13b
TRAF1: TNF receptor associated factor 1
TRIM25: Tripartite motif containing 25
TZDs: Thiazolidinediones
VAMP7: Vesicle associated membrane protein 7
VAMP7: Vesicle associated membrane protein 7
VEGF: Vascular endothelial growth factor
ZNFX1: Zinc finger NFX1-type containing 1
β-ACTIN: β actin
